# Characteristics of the Discoloration Switching Phenomenon of 4H-SiC Single Crystals Grown by PVT Method Using ToF-SIMS and Micro-Raman Analysis

**DOI:** 10.3390/ma17051005

**Published:** 2024-02-22

**Authors:** Seul-Ki Kim, Hajun Kim, Hyun Sik Kim, Tae Eun Hong, Younki Lee, Eun Young Jung

**Affiliations:** 1 Semiconductor Materials Center, Korea Institute of Ceramic Engineering and Technology, Jinju 52851, Republic of Korea; skkim@kicet.re.kr; 2 Department of Materials Engineering, Gyeongsang National University, Jinju 52828, Republic of Korea; 3 Department of Materials Engineering and Convergence Technology, Gyeongsang National University, Jinju 52828, Republic of Korea; khj42021@gmail.com; 4 Analysis and Standards Center, Korea Institute of Ceramic Engineering & Technology, Jinju 52851, Republic of Korea; hyunkim@kicet.re.kr; 5 Division of High-Technology Materials Research, Korea Basic Science Institute, Busan 46742, Republic of Korea; tehong@kbsi.re.kr; 6 The Institute of Electronic Technology, College of IT Engineering, Kyungpook National University, Daegu 41566, Republic of Korea

**Keywords:** 4H-SiC single crystal, physical vapor transport (PVT), discoloration switching, ToF-SIMS, Raman spectroscopy

## Abstract

The discoloration switching appearing in the initial and final growth stages of 4H-silicon carbide (4H-SiC) single crystals grown using the physical vapor transport (PVT) technique was investigated. This phenomenon was studied, investigating the correlation with linear-type micro-pipe defects on the surface of 4H-SiC single crystals. Based on the experimental results obtained using time-of-flight secondary ion mass spectrometry (ToF-SIMS) and micro-Raman analysis, it was deduced that the orientation of the 4H-SiC c-axis causes an axial change that correlates with low levels of carbon. In addition, it was confirmed that the incorporation of additional elements and the concentrations of these doped impurity elements were the main causes of discoloration and changes in growth orientation. Overall, this work provides guidelines for evaluating the discoloration switching in 4H-SiC single crystals and contributes to a greater understanding of this phenomenon.

## 1. Introduction

Silicon carbide (SiC) has attracted significant attention in recent years owing to its outstanding electrical, mechanical, and thermal properties. As a result, SiC is commonly employed in many fields due to its incorporation into power devices and optoelectronics [1,2,3,4,5]. Over 200 SiC polytypes are known to exist, with cubic (3C-SiC) and hexagonal (4H-SiC or 6H-SiC) −modified compounds being the most commonly used; these structural differences significantly influence the electrical properties and potential applications of each polytype. Among the various SiC polytypes, the cubic (3C-SiC) and hexagonal (4H-SiC or 6H-SiC) structures are the most commonly studied and used [2,6,7].

For example, 4H-SiC is known to exhibit a high-breakdown electric field at room temperature, in addition to high thermal conductivity and a wide bandgap [8,9]. A number of SiC polytypes also exist that exhibit high process temperatures and chemical stabilities, among which 4H-SiC is the most widely used in power electronic applications [10]. Commercial SiC may be classified as either conductive SiC or high-purity semi-insulating SiC. In the case of high-purity semi-insulating SiC, almost no impurity dopant atoms are present, thereby accounting for the colorless nature of 4H-SiC, which is treated to remove all impurities. More specifically, commercial 4H-SiC is colorless due to its wide band gap, which prevents absorption over the wavelength range of visible light. However, upon doping conductive SiC with N, P, Al, or B to generate additional energy levels [11], absorption occurs in the visible light region. In addition, doped SiC crystals are known to exhibit unique color characteristics, wherein the SiC crystal color becomes darker upon increasing the concentration of impurity elements [12]. Moreover, 4H-SiC single crystals are mainly grown using the slow physical vapor transport (PVT) approach [13,14], which leads to different crystal colors in the initial and final states of crystal growth. With these considerations in mind, it is clearly important to establish an analytical approach for monitoring this color change and to determine the mechanism behind this phenomenon in 4H-SiC crystals under different impurity concentrations. This is of particular importance in the context of process control and failure analysis for the obtained SiC crystals. 

Thus, to investigate the structural properties and spatial distributions involved in the discoloration of 4H-SiC single crystals, discoloration switching is performed, and the formation of linear-type micro-pipe defects is evaluated during two different growth periods, namely the initial (Case I) and final (Case II) growth periods. Considering these two growth periods, the characteristics of the 4H-SiC single crystals obtained by PVT are examined using gas discharge mass spectrometry (GDMS), micro-Raman spectroscopy, X-ray diffraction (XRD), photoluminescence (PL) spectroscopy, magnetic sector secondary-ion mass spectrometry (magnetic sector-SIMS), and time-of-flight secondary ion mass spectrometry (ToF-SIMS).

## 2. Materials and Methods

### 2.1. Preparation of 3C-SiC as a Precursor Material

The 3C-SiC (cubic silicon carbide) powder was synthesized in a customized furnace via a chemical vapor deposition (CVD) process, which included vaporization, pyrolysis, nucleation, oxidation–reduction, and substitution. Subsequently, it was reacted with granules at a high temperature [15]. The precursor gas comprised commercial methyltrichlorosilane (MTS) as the silicon precursor, as well as ammonia (NH_3_) and carbon dioxide (CO_2_). Ethylene (C_2_H_4_) and propane (C_3_H_8_) were used as hydrocarbon precursors due to their easy decomposition ability under high-temperature conditions. 

A mixture of H_2_ and Ar was employed as the carrier gas. The color of the synthesized 3C-SiC was black, indicating the presence of impurities or defects in the material. The 3C-SiC powder was crushed and milled using specially developed equipment to minimize contamination. The residual acid salt was removed by volatilization, and any excess oxygen present in the SiC was removed through denitrification under heating at 105 °C under reduced pressure in an argon environment. All other impurities present in the synthesized SiC were eliminated through the decarburization method by heat treatment at 850 °C for 1 h under oxygen atmosphere conditions to give a dried 3C-SiC powder with a purity of >90% [15,16]. The method for removing excess oxygen present in 3C-SiC powder was explained in detail as follows. There are some impurities in the 3C-SiC powder synthesized by the CVD process, such as excess oxygen, free carbon, and free silicon compounds (F-Si or F-SiO_2_). These by-products were removed through acid leaching, decarburization, and esterification. Firstly, the free silicon compounds were eliminated through acid leaching purification by using the mixed hydrochloric acid solution with a sodium catalyst. Thereafter, in the case of decarburization purification, the 3C-SiC powder was treated through thermal treatment by passing oxygen gas in order to remove CO_2_.

Then, the residual oxygen was purified by the OH substitution of acid through esterification at 105 °C on a hot plate [15,16]. Subsequently, the obtained powder was sieved through a 100 µm mesh to remove the fine and coarse particles. The synthesized 3C-SiC powder was also subjected to chemical pretreatment to remove any impurities introduced during the synthesis. 

**Figure 1:**
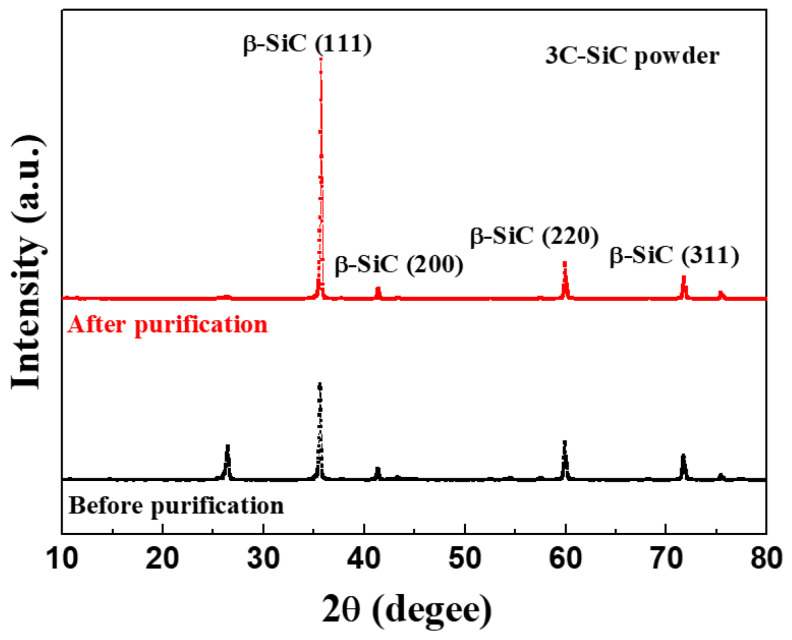
X-ray diffraction (XRD) patterns of the purified 3C-SiC powder as a starting material for 4H-SiC single crystals.

The elemental composition of the 3C-SiC powder was obtained using gas discharge mass spectrometry (GDMS), as listed in Table 1, and a purity of 99.999% was confirmed. The XRD patterns of the 3C-SiC powder showed peaks corresponding to 3C-SiC (β-SiC phase), as shown in Figure 1. The peaks at 35.7°, 41.2°, 59.9°, and 71.6° are attributed to the (111), (200), (220), and (311) planes of the β-SiC phase, respectively [15,16,17,18].

### 2.2. Growth of 4H-SiC Single Crystals Using the PVT Process

**Figure 2:**
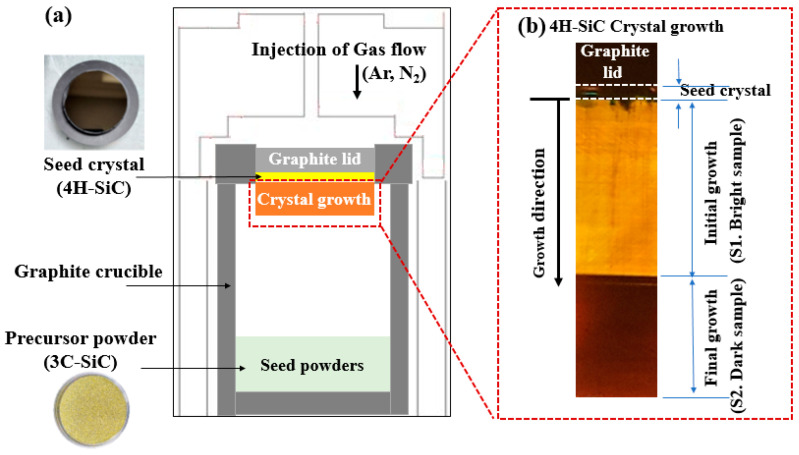
(**a**) Experimental set-up for the PVT process, and (**b**) a cross-sectional diagram for 4H-SiC single crystal growth.

**Figure 3:**
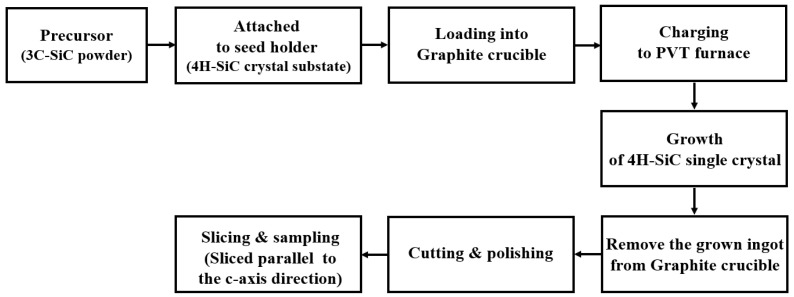
Detailed experimental procedure for growth of the 4H-SiC single crystals using the PVT process.

The 4H-SiC single crystals were grown from the 3C-SiC source powder using PVT [15], as outlined in Figure 2a and Figure 3. To grow 4H-SiC single crystals, the 3C-SiC powder was placed on the graphite crucible in the PVT reactor chamber. Crystal growth was conducted under an inert gas environment, wherein a 4H-SiC crystal ingot was grown by sublimation of the powder in the heated PVT reactor crucible. Detailed experimental conditions for the growth of the 4H-SiC single crystals using PVT as shown in Table 2.

More specifically, the growth of a single crystal was conducted under high-temperature conditions of 2300 °C and a pressure of 35 Torr in the presence of an argon/nitrogen gas mixture (99.96% purity). The injected argon gas flow rate was controlled using a mass flow meter. To maintain a stable gas pressure of 35 Torr during crystal growth, a mechanical pump was adjusted according to the required argon gas flow rate. The seed lid was attached to the top of the graphite crucible containing the 4H-SiC substrate. Crystal growth was allowed to proceed over 30 h with an average growth rate of ~1400 μm/h. After completion of the growth and cooling stages, the 4H-SiC crystal ingot was detached from the graphite crucible and sliced parallel to the c-axis direction. Subsequently, the sliced 4H-SiC was polished and cut perpendicular to the c-axis for analysis. Figure 2b shows a cross-sectional diagram of the 4H-SiC single crystal’s growth. The elemental composition of the grown 4H-SiC single crystals was obtained using GDMS (see Table 1), and a purity of 99.999% was confirmed.

**Figure 4:**
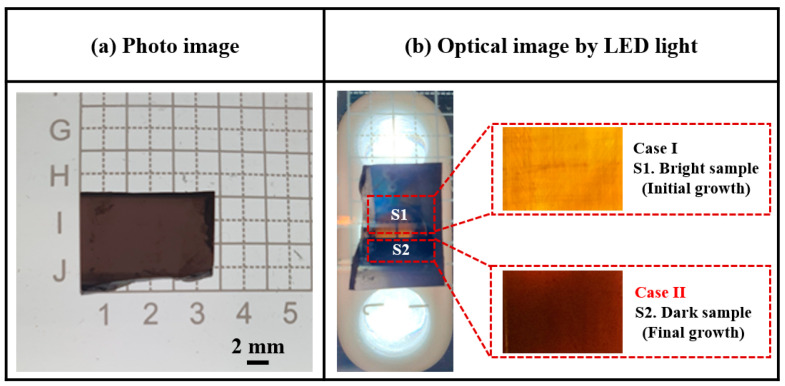
(**a**) Photographic image of the sliced 4H-SiC single crystal, and (**b**) optical images recorded under LED illumination for cases I and II.

Figure 4a,b presents photographic and optical images (under light-emitting diode (LED) illumination), respectively, recorded for the surface of a sliced 4H-SiC single crystal. Under LED illumination, the sliced 4H-SiC single crystal appeared to be divided into two color regions, namely Case I (bright, near side) and Case II (dark, external side). Detailed experimental conditions and parameters for the identification of the sliced 4H-SiC single crystals are presented in Table 3. 

### 2.3. Analysis and Characterization

The elemental compositions of the prepared 3C-SiC powder and the grown 4H-SiC single crystal were determined using gas discharge mass spectrometry (GDMS; Element GD Plus GD-MS, Thermo-Fisher Scientific, Waltham, MA, USA). Prior to the measurement, each sample was treated to produce a flat surface through sequential grinding, cutting, and polishing. An ionization power range of 30–50 W was applied for the trace element analysis.

The crystal orientations of the 4H-SiC single crystal were evaluated using multifunction X-ray diffraction (XRD; PANalytical, Malvern, UK) and high-resolution two-dimensional (2D) XRD (D8 Discover, Bruker, MA, USA) at the Korea Basic Science Institute (KBSI, Daegu, Republic of Korea). To acquire the diffraction planes for the major and minor XRD peaks, XRD spectra were measured over the θ–2θ range using multifunction XRD with Cu Kα radiation at 30 mA and 40 kV.

The structural phase and preferential orientation of all samples were performed using a Raman spectrometer (Renishaw, Wotton-under-Edge, UK) with a 514 nm laser as the excitation source. The Raman spectra detection was acquired over the wavenumber range of 200–2000 cm^−1^ with a four-stage Peltier-cooled CCD detector (UV-Vis-NIR range). The objective lens of the microscope (DM500, LEICA, Wetzlar, Germany) had a magnification of 50×, and the exposure time for accumulation was 5 s. The power intensity of the laser beam was 5.0 ± 0.1 mW in the exposure time for accumulation, which was 5 s. 

The photoluminescence (PL) spectra were obtained at both 298 K and 50 K using a PL spectrometer (LabRAM HR Evolution, Horiba, Kyoto, Japan) equipped with a He–Cd laser as the excitation source (wavelength = 325 nm, power = 0.15–15 mW). The power density of the used laser ranged from 0.023 to 23.6 kW cm^−2^ for measurement. The laser was focused on the sample using a 50× objective lens. 

To quantitatively monitor the elemental depth distribution in the 4H-SiC single crystal, magnetic sector secondary-ion mass spectrometry (magnetic sector-SIMS; IMS-6f, Cameca, France) was employed. For the magnetic sector-SIMS measurements, the ^12^C^14^N^−^ secondary ions were acquired using a cesium ion (15 kV), while the ^11^B^+^ and ^27^Al^+^ secondary ions were acquired using oxygen ions (7.5 kV) at a high mass resolution. Oxygen (^32^O_2_^+^, 10 keV) was applied as the sputtering ion source for the detection of the secondary ions. The primary ion was rastered over an area of 200 × 200 μm^2^, and the secondary ion signal was recorded from the central part of this area (~60 μm diameter). After the magnetic sector-SIMS depth profiling was completed, the formed crater depth was measured using a stylus profiler. A constant erosion rate was assumed for the conversion of the sputtering time to the corresponding depth. 

To analyze the component distributions on the micro-pipe defect surfaces of the Case I and Case II 4H-SiC crystal samples, time-of-flight secondary ion mass spectroscopy (ToF-SIMS; IonTOF 5, ION-TOF, Münster, Germany) was used to measure both the positive and negative-ion modes under high-current bunching conditions. The pressure in the analysis chamber was maintained at a very low vacuum level below 1 × 10^−9^ Torr. The voltage and Bi_1_^+^ ion current were 30 kV and 1 pA, respectively. The negative-ion and positive-ion mass spectra were acquired from a 500 × 500 µm^2^ area using a Bi_1_^+^ (1 pA) primary ion beam operating at 30 keV. The mass resolution of the measured spectra was measured under high-resolution conditions of 8000 or more at a mass-to-charge ratio (*m*/*z*) of ^29^Si. To prevent surface charging during the ToF-SIMS measurements, a flood gun of low-energy electrons was used. 

To further analyze the component distributions on the micro-pipe defect surfaces, ToF-SIMS images were recorded using the burst alignment mode, with Bi_3_^+^ primary ions operating at a voltage and ion current of 30 keV and 0.6 pA, respectively. The ToF-SIMS images were acquired over an area range of 500 × 500 µm^2^ in both the negative-ion and positive-ion modes. 

In addition, to investigate the vertical distributions of the components in the depth direction for the Case I and Case II samples, the sputter raster was set at 150 × 150 mm^2^, and the secondary ions were detected using ToF-SIMS with a depth profile mode based on an area of 50 × 50 mm^2^ centered within the sputtered region. A pulsed electron flood source was employed for charge compensation.

For the negatively charged secondary ions, the depth profile was measured using Cs^+^−sputtering ions operating at a voltage and ion current of 3 keV and 37 nA, respectively. The depth profile of the positively charged secondary ions was measured in the non-interlaced analysis mode using O_2_^+^ primary ions at a voltage and ion beam current of 2 keV and 310 nA, respectively. 

## 3. Results

**Figure 5:**
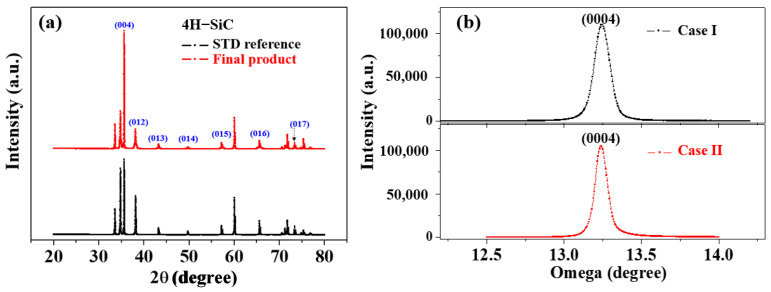
(**a**) XRD patterns and (**b**) X-ray rocking curves of Case I and Case II 4H-SiC single crystals in the (0004) direction.

Figure 5a,b shows the XRD patterns and X-ray rocking curves of the Case I and Case II 4H-SiC single crystals in the (0004) direction, wherein it can be seen in Figure 4a that all samples prepared by PVD were preferentially grown along strong (004) planes. In this figure, the XRD peaks observed at 35.6°, 38.1°, 43.2°, 49.7°, 57.2°, 65.7°, and 74.9° were attributed to the (004), (012), (013), (014), (015), (016), and (017) planes of the 4H-SiC phase, respectively [15,16,19]. All peaks were in good agreement with ICSD card 98-016-4971. In addition, the full width at half-maximum (FWHM) values for the two (0004) X-ray rocking curves exhibit similar values below 0.01°, thereby indicating that both 4H-SiC specimens are highly crystalline. 

**Figure 6:**
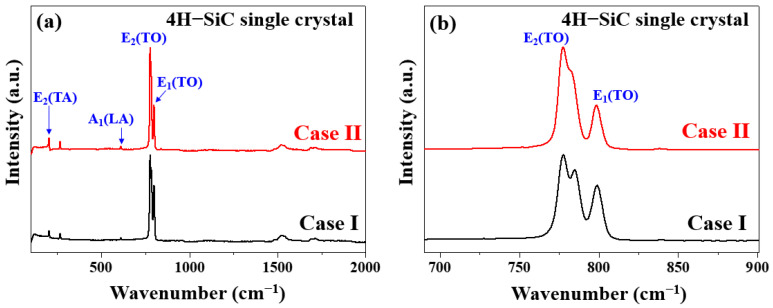
Raman spectra of the two 4H-SiC single crystals (cases I and II) measured under (**a**) wide and (**b**) narrow scanning conditions for the TO peaks.

The Raman spectra of the grown 4H-SiC crystals are shown in Figure 6a,b, wherein the two characteristic peaks of 4H-SiC, corresponding to the transverse optical (TO) phonon, were detected at ~778 and 797 cm^−1^ [5,20,21,22,23,24]. For the Case I (bright) crystals, the TO peak observed (FTO, E_2_) at 781 cm^−1^ in the perpendicular orientation was split into a doublet at 777 and 783 cm^−1^, indicating that the 4H-SiC structure of the single crystal did not change, but the preferred orientation of the (0001) plane changed [24,25]. In Case II (dark), the TO peak (FTO, E_2_) was observed at 781 cm^−1^ in the parallel orientation, and no splitting was evident. In the early stages of growth (Case I), the FTO peak intensity initially weakened prior to strengthening again in the final growth stage (Case II) until the crystal’s growth was complete. The Raman spectra presented in Figure 6b for cases I and II were recorded with the incident laser oriented parallel to the c-axis.

Based on the obtained experimental results, it was apparent that all samples maintained the 4H-SiC structure [24]. For the Case II (dark) specimen, the folded TO peak (FTO, E_2_) recorded in the perpendicular orientation at 781 cm^−1^ was split into a doublet at 777 and 783 cm^−1^ [24]. Considering the displacement of atoms from their original positions in the lattice, it was evident that this splitting occurred perpendicular to the change in the rotational c-axis of the defect [24]. Table 4 summarizes the detailed assignment and origin of the Raman peaks with respect to three different sample conditions. This splitting occurred perpendicular to the change in the rotational defect to the c-axis, considering the displacement of atoms from their original positions in the lattice [24].

**Figure 7:**
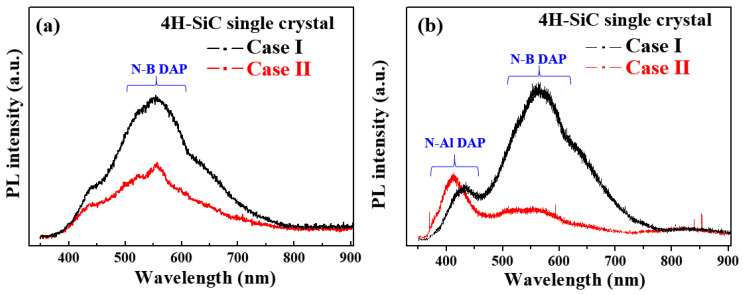
PL spectra of the 4H-SiC single crystals (cases I and II) measured (**a**) at room temperature (298 K) and (**b**) at a low temperature of 50 K in the wavelength range of 300–900 nm.

Figure 7a shows the PL emission spectra measured at room temperature (298 K) with a 325 excitation source for sample cases I and II. For all samples, the obtained PL spectra showed a wide non-gaussian symmetric peak at 533 nm. This PL peak corresponds to nitrogen–boron (N–B), which originated from the N–B donor–acceptor pair (DAP) emission [21,22]. The luminescence emissions displayed in these spectra are directly correlated with energy-level transitions of semiconductors. Thus, the recombination of DAPs in the 4H-SiC crystals containing an indirect bandgap leads to the formation of free excitons and phonons. This type of recombination introduces a complex donor-acceptor recombination mechanism, indicating the potential of the impurity concentration to impart a critical influence on the luminescence emissions [26,27,28]. 

Figure 7b shows the PL spectra measured at a low temperature of 50 K for specimen cases I and II. In all samples, the nitrogen–aluminum (N–Al) and N–B DAP emissions were also detected and were confirmed at 420 and 580 nm, respectively [26,27,28], with the highest peak intensity being observed for the N–Al DAP emission of Case II. In addition, the N–B DAP emission peak intensity was high for Case I, suggesting an increase in the N–B DAP density at low temperatures [26,27,28]. Moreover, the peak intensity of the N–B DAP emission increased with an increasing nitrogen concentration. As shown in Figure 7b, Case I (bright) exhibits a weak PL peak at 370 nm in the low-temperature spectrum, which was attributed to the nitrogen emission caused by nitrogen-bounce excitation [26,27,28]. In contrast, the N–Al DAP emission was observed at 420 nm in Case II due to the effect of a visible emission at 533 nm that was caused by luminescence quenching [26,27,28].

**Figure 8:**
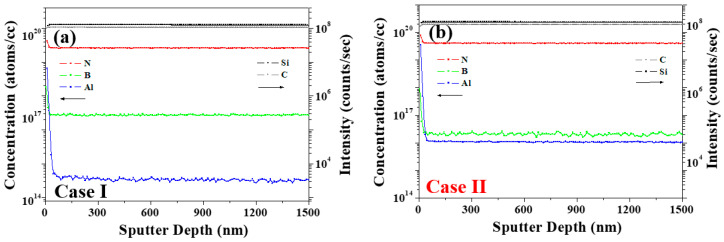
Depth profiling results obtained by using a magnetic sector-SIMS with two different samples of cases (**a**) I and (**b**) II.

To better clarify the correlation between the impurities and the PL behavior, magnetic sector-SIMS depth profiling was performed to identify the trace impurity concentrations of B, Al, and N with two different samples of cases (a) I and (b) II. As outlined in Figure 8 and Table 5, the concentration of each impurity was converted into atoms cm^−3^, and the donor–acceptor recombination ratios (RDAs) of C_D−A_ and 2C_B_/(C_N_ − C_B_) were calculated using Equation (1) [15,21,22], wherein the B, Al, and N concentrations are defined as C_B_, C_Al_, and C_N_, respectively. The parameter C_D−A_ was calculated as a function of C_B_ − (C_Al_ − C_N_) [16] to give the values listed in Table 5.

C_D−A_ = ln C_B_ − (ln C_Al_ − ln C_N_), (1)

In Case II (dark), the increase in the PL intensity of the N–B DAP originates from an increase in the RDA of C_D−A_ [15]. Considering that the DAP recombination rate is an efficient measure of the emission luminescence [29,30,31,32], and the RDA is proportional to the donor concentration (C_D_) and the acceptor concentration (C_A_), the correlation between the C_D_, C_A_, and PL properties can be evaluated, as described previously [21]. The increased N–B DAP peak intensity for Case II was, therefore, attributed to an increase in C_N_. Aukerman and Millea’s model [30,31,32] suggests that the correlation between DAP recombination and concentration causes the PL intensity to increase with an increase in the difference between C_N_ and C_B_; this is known as the N–B concentration gap. More specifically, when the N–B concentration gap is larger than C_B_, saturation occurs, whereas when the N–B concentration gap is more than twice C_B_, the PL emission intensity decreases due to the presence of non-radiative defects (non-emission), as evidenced in Case II [29]. For Case II, at a high Al concentration (C_Al_), the N–B and N–Al DAP emissions at 420 nm became weaker [15]. As shown in Figure 8 and Table 5, for Case I, the concentrations of B, N, and Al were calculated to be 1.29 × 10^17^, 3.49 × 10^19^, and 5.49 × 10^14^ atoms cm^−3^ respectively, whereas in Case II, the corresponding concentrations were 1.99 × 10^16^, 4.04 × 10^19^, and 1.05 × 10^16^ atoms cm^−3^, respectively. From these results, it is clear that the concentrations of N and Al were higher in Case II than in Case I. The 2C_B_/(C_N_ − C_B_) value was calculated to compare the correlation between the N–B concentration and PL [15,29]. 

For all samples, the peak intensity of the N–B emission at 580 nm decreased in the PL spectra, as shown in Figure 7a,b. It indicates that the N impurity has been substituted with carbon atoms within the SiC lattice; that is, these substitutions trapped the impurity levels within the bandgap of the material, and then, the impurity levels act as trap states for charge carriers (electrons and holes) and make it difficult for them to transition between the impurity levels to a non-radiative emission of 580 nm owing to the electron–hole pair recombination, resulting in discoloration and luminescence quenching. 

For the Case II sample, the calculated value of 2C_B_/(C_N_ − C_B_) was 0.001 less than 0.01, meaning a weak visible emission at a wavelength of 580 nm. The presence of Al and N impurities in the hexagonal lattice of the SiC results in a blue emission peak that is derived from N–B DAP emission quenching. This emission peak means that the impurity concentrations of N, B, and Al have an influence on N–B DAP emission quenching in 4H-SiC [15]. 

**Figure 9:**
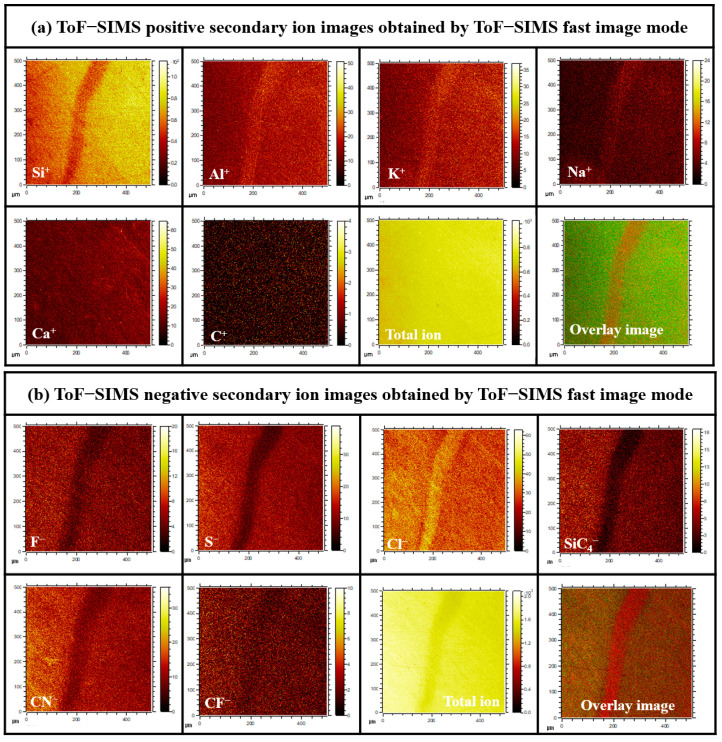
(**a**) Positive and (**b**) negative secondary ion ToF-SIMS images recorded in the fast image mode for the Case I (bright) sample.

To investigate the linear-type micro-pipe defects of the 4H-SiC surface, the different surface component distributions were analyzed using ToF-SIMS for the Case I 4H-SiC single crystal. Figure 9a,b shows the ToF-SIMS images obtained for the micro-pipe region of the crystal using the ToF-SIMS fast-imaging mode. For Case I, strong Na^+^, Al^+^, and K^+^ peaks were detected in the micro-pipe defects using the positive-ion mode (Figures S1 and S2), whereas Cl^−^ was detected with high intensity in the negative-ion mode (Figures S3 and S4). It was therefore confirmed that the impurities of Na, Al, K, and Cl were highly distributed in the micro-pipes defect region of Case I’s surface. However, no micro-pipe defects were observed on Case II’s surface due to the expected distribution of impurities throughout the SiC species. It was considered that this occurred via diffusion at the high-temperature conditions employed during the final stage of crystal growth (Figure S5). 

**Figure 10:**
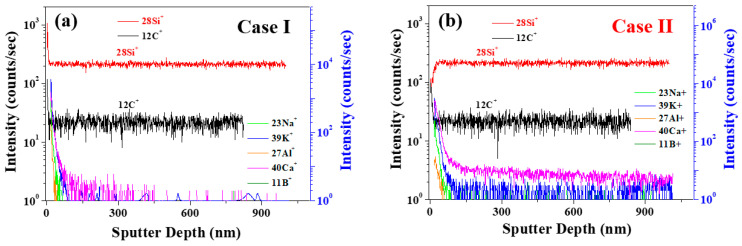
ToF-SIMS positive-ion depth profiling of 4H-SiC single crystal specimens with two different samples of cases (**a**) I and (**b**) II.

To clarify the different spatial distributions in the depth direction, ToF-SIMS depth profiling was performed for Case I and Case II’s 4H-SiC single crystal specimens, as presented in Figure 10a,b. More specifically, from Figure 10a, it can be seen that the impurities of Case I were distributed only on the SiC surface, compared to Case II (Figures S1 and S2). These results are consistent with the fast image results presented in Figure 9. Thus, the Na, K, Al, and Ca impurities penetrated SiC and were distributed throughout the structure for Case II, as shown in Figure 10b. 

**Figure 11:**
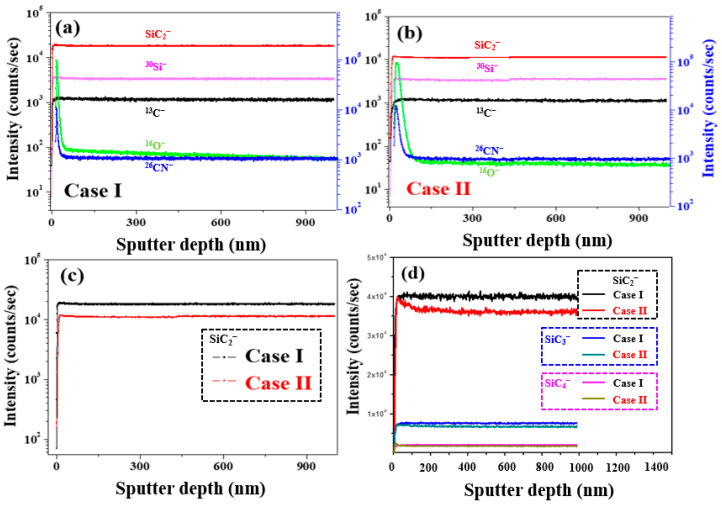
ToF-SIMS negative-ion depth profiling for the 4H-SiC single crystal conditions: (**a**) Case I, (**b**) Case II, and (**c**) SiC_2_^−^. (**d**) Comparison of the SiC_2_^−^, SiC_3_^−^, and SiC_4_^−^ ToF-SIMS depth profiling results.

Figure 11a,c shows the ToF-SIMS negative-ion depth profiling results of 4H-SiC single crystal specimens with respect to the Cases I and II. More specifically, as shown in Figure 11c, the highest SiC_2_^−^ content was detected in Case I, indicating that during this initial stage of crystal growth, the specimen is richer in carbon than during the latter stages (i.e., Case II). In Figure 11d, it can be seen that this higher carbon content can be attributed to an increase in SiC_2_. It was therefore considered that this carbon species may be associated with the micro-pipe defects observed in the Case I specimen. Indeed, during the PVT process, the 3C-SiC precursor powder was sublimated to generate vaporized species, such as Si, Si_2_C, and SiC_2_, which exist in a silicon-rich and carbon excess, as indicated in Equation (2) [33,34,35]:

SiC (s) → Si (g) + Si_2_C (g) + SiC_2_ (g) + C (s) (2)

Since these vapor species are initially desorbed during the growth process, the surface of the grown SiC ingot becomes carbon-rich, as shown in Figure 11. At this time, when a metal cation with a large atomic radius, such as Ca, comes into contact with the C or Si-rich species, impurity substitution in the SiC crystal lattice can lead to the generation of stress or discoloration [33,34,35]. We suggested a method to reduce impurity substitution. Firstly, in the case of the metal impurities, the metal soluble acid salts (MCl _3_ and MNO_3_) in the 3C-SiC powder were purified through a catalytic reaction using hydrochloric acid and esterification (CH_3_ONO + H_2_O) [15,16]. Secondly, in the case of the effect of the C/Si ratio related to the N impurity, when the C/Si ratio increases, the N impurities incorporation into a 4H-SiC crystal can be reduced by site competition rules [35]. Additionally, to maintain the C-rich conditions, the growth surface of the 4H-SiC seed was placed on the C face, in which the N molecule was very weakly adsorbed.

## 4. Conclusions

This work systematically investigated the main factors of discoloration switching for 4H-SiC single crystals grown using the physical vapor transport (PVT) technique. For this purpose, time-of-flight secondary ion mass spectrometry (ToF-SIMS) and micro-Raman analyses were employed. Considering the crystal structure and the distribution of trace elements, the origin of the color changes between the early and late stages of crystal growth. The ToF-SIMS results showed that in the early stages (Case I), the bright color was attributed to some formation of micro-pipe linear defects on 4H-SiC’s single crystal surface during crystal growth. In the latter stages (Case II), a dark color was observed, and no micro-pipe defects were found. These differences were attributed to the abundance of SiC_2_ in the initial stage and changes in the C/Si ratio as the crystal growth proceeded. Furthermore, the disappearance of micro-pipe defects in the final growth stage was likely due to the penetration and dispersion of any metal impurities inside the SiC matrix under the high-temperature conditions employed during this stage. The discoloration defects, therefore, originated from the presence of trace impurities and induced changes in the preferred orientation of 4H-SiC. Moreover, it was considered that the observed changes in the visible green light luminescence were affected by luminescence quenching during the latter growth stages. Overall, this work provides guidelines for evaluating the defect characteristics of PVT-grown 4H-SiC single crystals, with the aim of improving crystal homogeneity.

## Figures and Tables

**Table 1:** Detailed element compositions of the 3C-SiC powder and the grown 4H-SiC single crystals, as determined using gas discharge mass spectrometry (GDMS).

Element (ppm)	B	Al	P	Na	V	Ti	Fe	Ca	Cl	S
3C-SiC raw powder	1.10	0.08	<0.05	0.16	<0.05	<0.05	0.34	<0.50	1.1	0.14
4H-SiC crystal	1.20	0.10	<0.05	0.10	<0.05	<0.05	<0.10	<0.50	0.71	0.07

**Table 2:** Detailed experimental conditions for the growth of the 4H-SiC single crystals using PVT.

Precursor material	3C-SiC polycrystalline powder
Reaction gas	Mixture of argon and nitrogen gas
Working pressure	35 Torr
Growth temperature	2300 °C
Growth rate	1400 μm/h
Growth time	30 h
Two case studies	Case I: Bright sample
	Case II: Dark sample

**Table 3:** Detailed experimental conditions and identification parameters for the sliced 4H-SiC single crystal sample regions corresponding to cases I and II.

Sample Identification	Condition	Collection Position	Photographic Image
Case I	S1. Bright sample	Seed near side of 4H-SiC Ingot	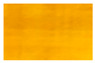
Case II	S2. Dark sample	External side of 4H-SiC Ingot	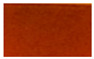

**Table 4:** Peak center positions of the Raman peaks of cases I and II relative to the 4H-standard reference.

Sample Identification	Color	FTO, Transversal (Planar) Optic, E_2_	
		‖ (cm^−1^)	⊥ (cm^−1^)
Case I	Bright yellow	N.D.	777, 783
Case II	Dark brown	781	N.D.

**Table 5:** Comparison of the calculated elemental concentrations of B, N, and Al from the depth profiling obtained by magnetic sector-SIMS depth profiling with two different samples of cases I and II.

Concentration	Sample Conditions	
	Case I (S1, Bright)	Case II (S2, Dark)
C_N_ (atoms cm^−3^)	3.49 × 10^19^	4.04 × 10^19^
C_B_ (atoms cm^−3^)	1.29 × 10^17^	1.99 × 10^16^
C_Al_ (atoms cm^−3^)	5.49 × 10^14^	1.05 × 10^16^
Ln C_N_	45	45
Ln C_B_	39	38
Ln C_Al_	34	37
RDA of C_D−A_ (arb. units)	50	46
The ratio of 2C_B_/(C_N_ − C_B_)	0.01	0.001

## Data Availability

Data are contained within the article and Supplementary Materials.

## Supplementary Materials

The following supporting information can be downloaded at: https://www.mdpi.com/article/10.3390/ma17051005/s1, Figure S1: ToF-SIMS positive secondary ion spectra recorded over two different regions: (a) ROI-1 and (b) ROI-2. The spectra were acquired from the ToF-SIMS positive secondary ion images obtained using the ToF-SIMS fast imaging mode for Case I (bright); Figure S2: Comparison of the normalized ToF-SIMS peak intensities of the ToF-SIMS positive secondary ion spectra recorded over two different regions (ROI-1 and ROI-2) and acquired from ToF-SIMS positive secondary ion image obtained using the ToF-SIMS fast imaging mode for Case I (bright); Figure S3: ToF-SIMS negative secondary ion spectra recorded over two different regions: (a) ROI-1 and (b) ROI-2. The spectra were acquired from the ToF-SIMS positive secondary ion images obtained using the ToF-SIMS fast imaging mode for Case I (bright); Figure S4: Comparison of the normalized ToF-SIMS peak intensities of the ToF-SIMS negative secondary ion spectra recorded over two different regions (ROI-1 and ROI-2) and acquired from ToF-SIMS positive secondary ion image obtained using the ToF-SIMS fast imaging mode for Case I (bright); Figure S5: ToF-SIMS (a) positive and (b) negative secondary ion images obtained using the ToF-SIMS fast imaging mode for Case II (dark) in the obtained 4H-SiC single crystal.
