# Supplementary material for: Characteristics of the Discoloration Switching Phenomenon of 4H-SiC Single Crystals Grown by PVT Method Using ToF-SIMS and Micro-Raman Analysis
Source: Materials (Basel). 2024 Feb 22;17(5):1005. doi: 10.3390/ma17051005 (PMC10934884; doi:10.3390/ma17051005)
Supplement: Supplementary file 1 [file materials-17-01005-s001.zip › materials-2850751-supplementary.pdf]

Supporting Information

Characteristics of the Discoloration Switching Phenomenon of 4H-SiC Single Crystals Grown by PVT Method Using ToF-SIMS and Micro-Raman Analysis

Seul–Ki Kim[1,2]*, Hajun Kim*[3]*, Hyun Sik Kim*[4]*, Tae Eun Hong*[5]*, Younki Lee*[3]**, and Eun Young Jung*[6]***

[1]Semiconductor Materials Center, Korea Institute of Ceramic Engineering and Technology, Jinju 52851, Republic of Korea

[2]Department of Materials Engineering, Gyeongsang National University, Jinju 52828, Republic of Korea

[3]Department of Materials Engineering and Convergence Technology, Gyeongsang National University, Jinju 52828, Republic of Korea

[4]Analysis and Standards Center, Korea Institute of Ceramic Engineering & Technology, Jinju 52851, Republic of Korea

[5]Division of High-Technology Materials Research, Korea Basic Science Institute, Busan 46742, Republic of Korea

[6]The Institute of Electronic Technology, College of IT Engineering, Kyungpook National University, Daegu 41566, Republic of Korea

*Correspondence

Younki Lee

Department of Materials Engineering and Convergence Technology, Gyeongsang National University, Jinju 52828, Republic of Korea

E-mail: ylee@gnu.ac.kr

Eun Young Jung

The Institute of Electronic Technology, College of IT Engineering, Kyungpook National University, Daegu 41566, Republic of Korea

E-mail: eyjung@knu.ac.kr

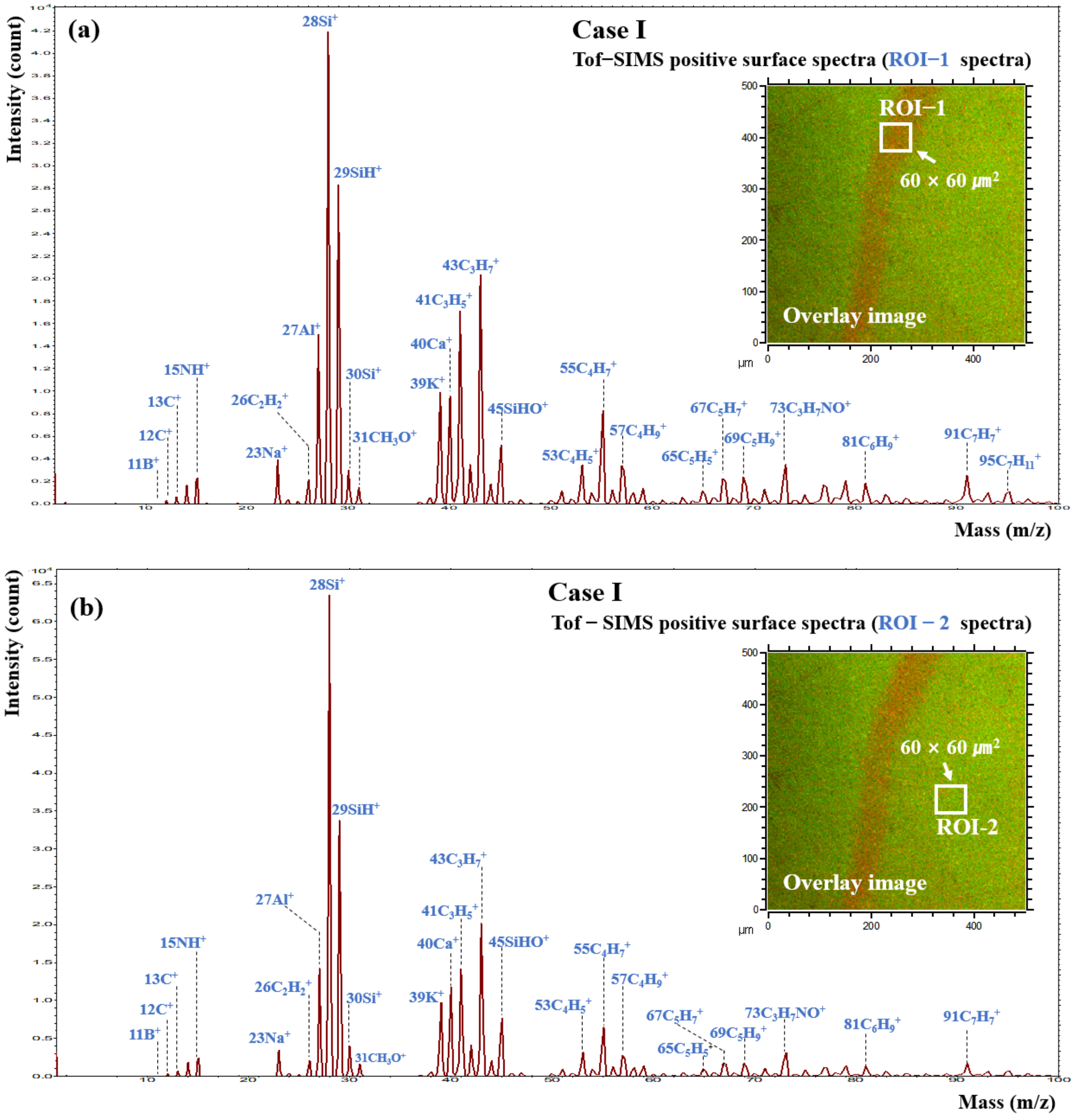

Figure S1: ToF−SIMS positive secondary ion spectra recorded over two different regions: (a) ROI−1 and (b) ROI−2. The spectra were acquired from the ToF−SIMS positive secondary ion images obtained using the ToF-SIMS fast imaging mode for Case I (bright).

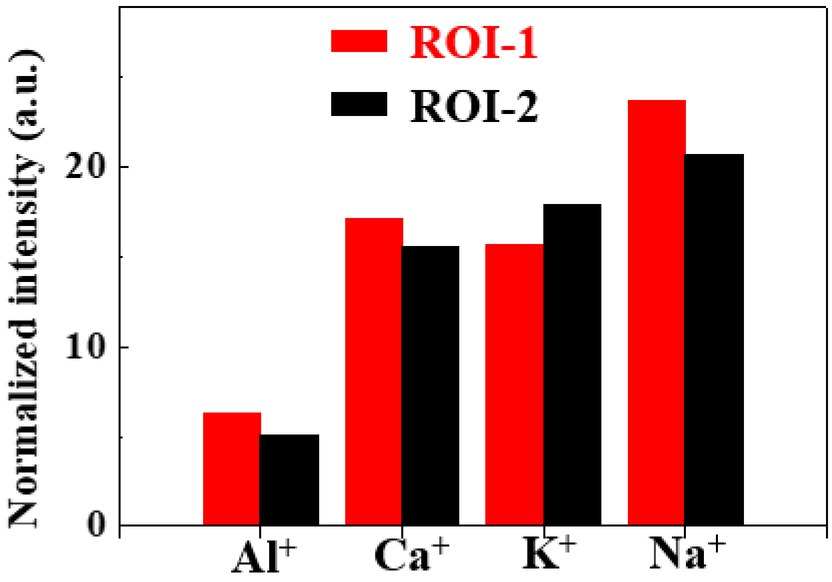

Figure S2: Comparison of the normalized ToF−SIMS peak intensities of the ToF−SIMS positive secondary ion spectra recorded over two different regions (ROI−1 and ROI−2) and acquired from ToF−SIMS positive secondary ion image obtained using the ToF-SIMS fast imaging mode for Case I (bright).

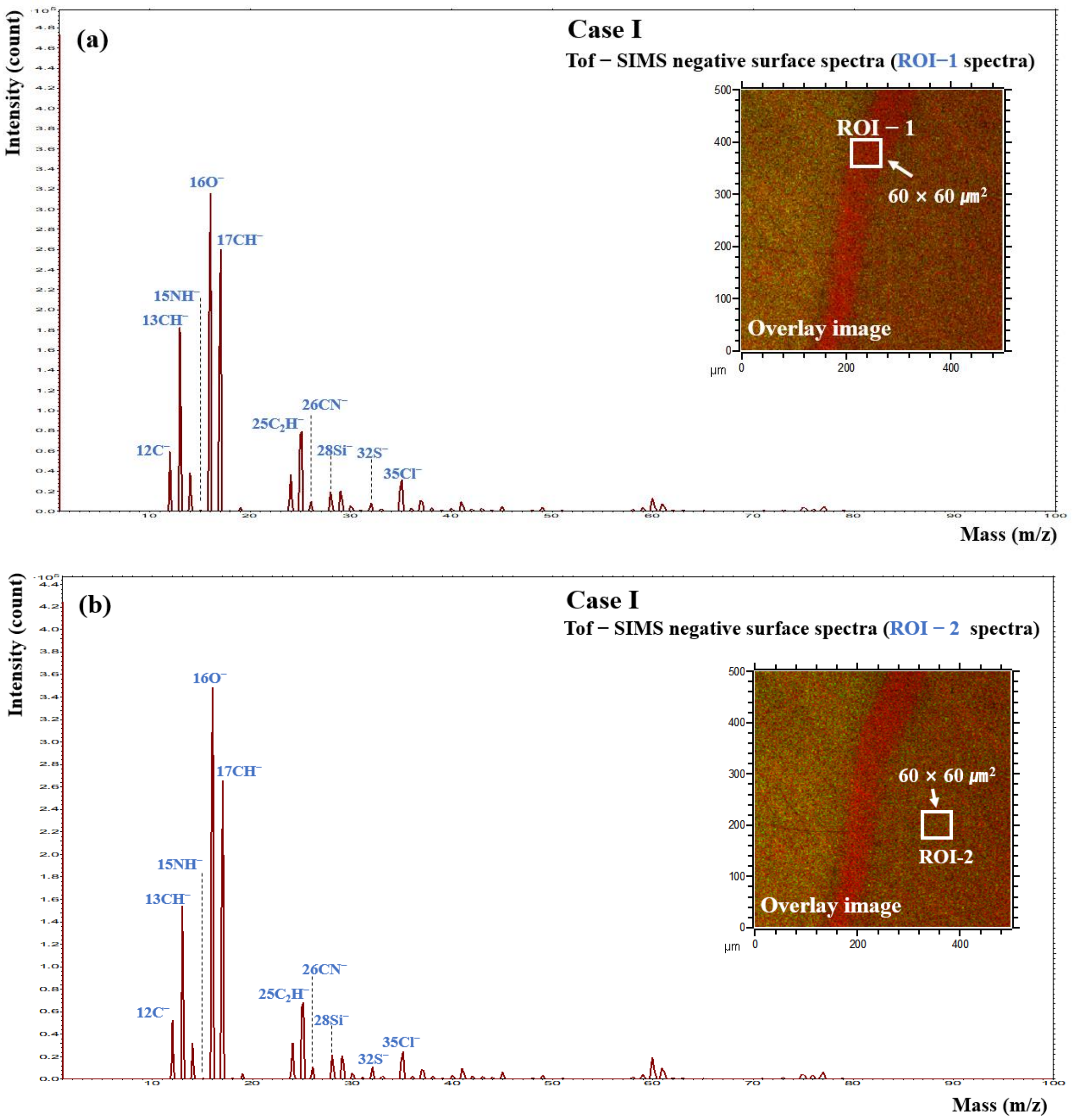

Figure S3: ToF-SIMS negative secondary ion spectra recorded over two different regions: (a) ROI−1 and (b) ROI−2. The spectra were acquired from the ToF−SIMS positive secondary ion images obtained using the ToF−SIMS fast imaging mode for Case I (bright).

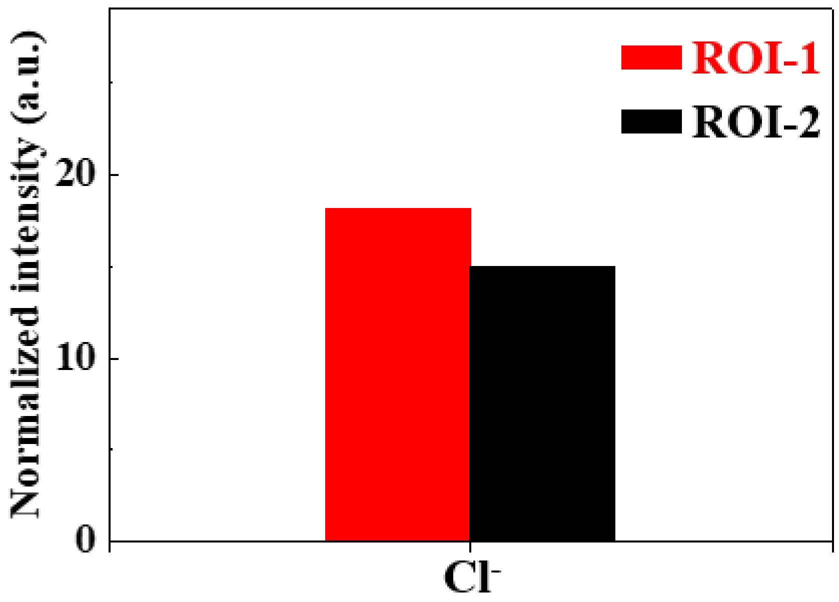

Figure S4: Comparison of the normalized ToF−SIMS peak intensities of the ToF−SIMS negative secondary ion spectra recorded over two different regions (ROI−1 and ROI−2) and acquired from ToF−SIMS positive secondary ion image obtained using the ToF−SIMS fast imaging mode for Case I (bright).

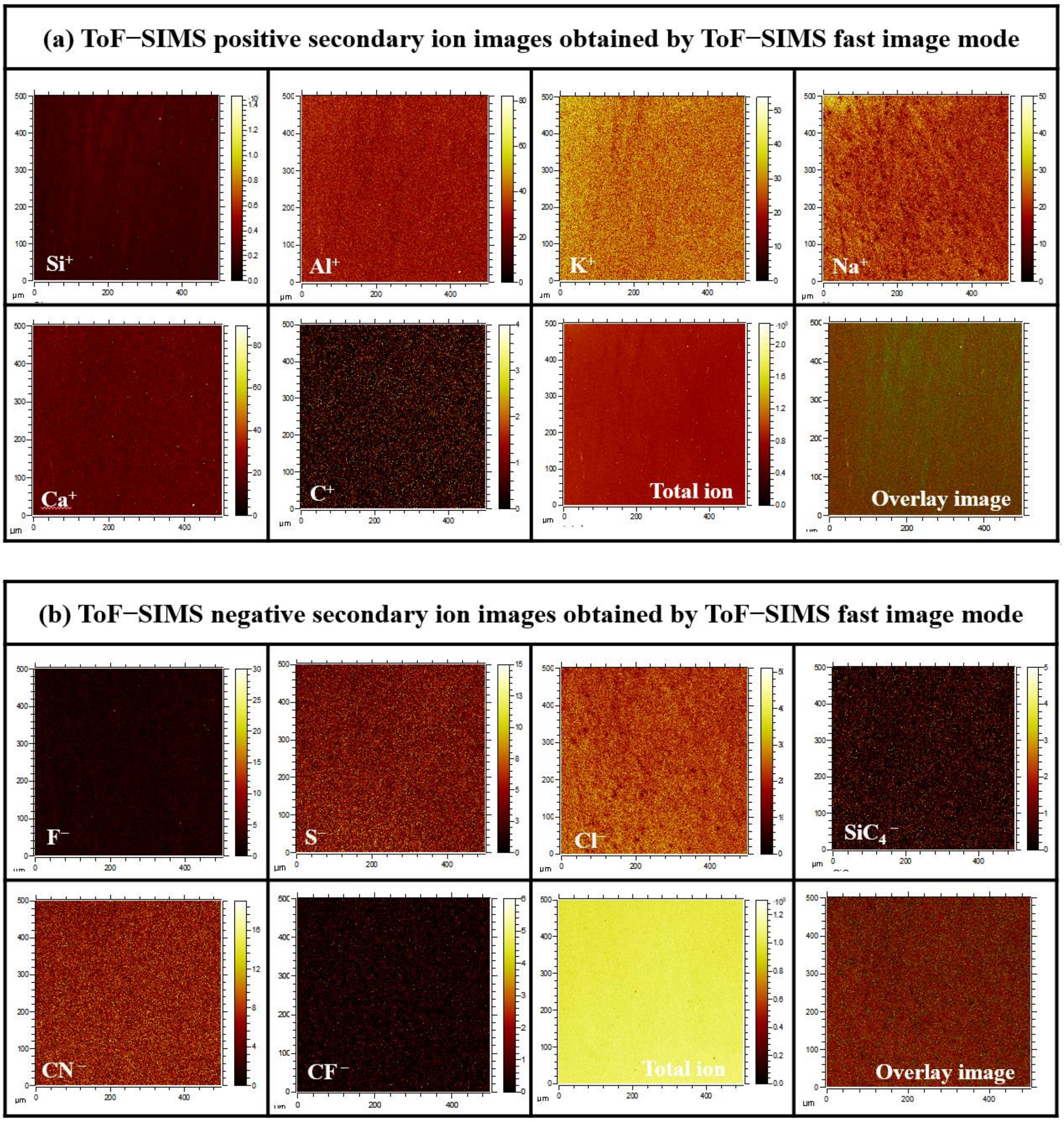

Figure S5: ToF−SIMS (a) positive and (b) negative secondary ion images obtained using the ToF−SIMS fast imaging mode for Case II (dark) in the obtained 4H−SiC single crystal.
